# Qiliqiangxin reduced cardiomyocytes apotosis and improved heart function in infarcted heart through Pink1/Parkin -mediated mitochondrial autophagy

**DOI:** 10.1186/s12906-020-02992-7

**Published:** 2020-07-02

**Authors:** Junyang Zhou, Zhixiao Wang, Yun He, Xinxia Luo, Wenjun Zhang, Li Yu, Xiuying Chen, Xiju He, Yahong Yuan, Xiaoli Wang, Xinrong Guo, Junming Tang, Mingan Zhu, Dongsheng Li, Yan Ding

**Affiliations:** 1 Hubei Key Laboratory of Embryonic Stem Cell Research, Taihe Hospital, Hubei University of Medicine, Shiyan, 442000 Hubei China; 2 Cardiovascular Department, Hubei University of Medicine, Taihe Hospital, Hubei University of Medicine, Shiyan, 442000 Hubei China; 3 Ultrasonography Department, Hubei University of Medicine, Taihe Hospital, Hubei University of Medicine, Shiyan, 442000 Hubei China; 4 Laboratory Department of the First Affiliated Hospital of Guizhou Medical University, Guiyang, 550000 Guizhou China; 5 Biomedical Research Institute, Hubei University of Medicine, Taihe Hospital, Hubei University of Medicine, Shiyan, 442000 Hubei China

**Keywords:** Qiliqiangxin, Myocardial infarction, Mitophagy, Heart function

## Abstract

**Background:**

Qiliqiangxin (QLQX) is a preparation refined from a traditional Chinese medicine compound. It plays an important role in protecting cardiac function after myocardial infarction (MI). However, the underline mechanism of QLQX action is not clear. The purpose of this study was to detect the effects of QLQX on mitophagy after MI.

**Methods:**

Male FVB/NJ mice aged 8–10 weeks were underwent left coronary artery ligation and were orally administered either QLQX (0.25 g/kg/d) or saline. Twenty-eight days after surgical operation, the cardiac function of mice was detected by echocardiography. Electron Microscopy was used to observe the microstructure of cardiomyocytes. Myocardial apoptosis was examined by TdT-mediated dUTP Nick-End Labeling (TUNEL) and western blot. H9c2 cells were cultured in a hypoxic incubator chamber (5% CO_2_, 1% O_2_, 94% N_2_) for 12 h and pretreated with or without QLQX (0.5 mg/mL). The cell apoptosis, reactive oxygen species (ROS), mitochondrial membrane potential and mitophagy were detected.

**Results:**

When compared to sham group, the cardiac function of MI mice decreased significantly, and their cardiomyocyte apoptosis and mitochondrial damage were more serious. These MI-induced cardiac changes could be reversed by QLQX treatment. In vitro experiments also confirmed that QLQX could protect cardiomyocytes from hypoxia-induced apoptosis and mitochondrial damage. Further study indicated that QLQX could increase the expression of Pink1 and Parkin in cardiomyocytes.

**Conclusion:**

Qiliqiangxin could reduce cardiomyocytes apotosis and improved heart function in infarcted heart through Pink1-mediated mitochondrial autophagy.

## Background

The incidence and mortality of myocardial infarction are still high in most countries in the world [1]. Although great progress has been made in the treatment of myocardial infarction, such as early reperfusion therapy, which has significantly reduced the mortality rate of myocardial infarction patients [2, 3]. However, at the same time, the morbidity and mortality of left ventricular remodeling and heart failure are increasing after myocardial infarction, which has become an increasingly worrying and challenging health problem [4, 5]. At present, treatment strategies for preventing left ventricular remodeling after myocardial infarction are still limited. Therefore, it is very necessary to conduct in-depth research on the pathological mechanism of myocardial injury and ventricular remodeling after myocardial infarction, and to determine additional treatment targets and treatment schemes to prevent the adverse effects of left ventricular remodeling after myocardial infarction [6].

As a central organ of the circulatory system, the heart needs tremendous amounts of energy to maintain its dynamic function, while mitochondria in cardiomyocytes are fundamental providers of cardiac energy. Mitochondrial malfunction and structural abnormalities are bound to cause myocardial damage and lead to cardiac systolic and diastolic dysfunction [7]. Recent studies have shown that in most eukaryotic cells, autophagy can be induced by nutrient deficiency, oxidative stress, salt stress or infection to remove damaged organelles, such as mitochondria, or to degrade normal organelles to reduce the operational burden thereby maintaining cell survival [8]. Autophagy occurs at different times and intensities, which may have different effects on tissue cells in different ways. Moderate autophagy can regulate the intracellular environment and maintain the stability of the intracellular environment, while excessive autophagy may lead to autophagic cell death and cause tissue damage. Mitophagy is a kind of selective autophagy [9]. It has attracted more and more attention because of its close relationship with the quantity and physiological function of mitochondria. Mitochondria provide the main energy for myocardial contraction-relaxation and control the key survival and death pathways in cells. Therefore, it plays an important role in maintaining cardiac homeostasis.

Some natural medicines are very effective in certain diseases [10–16]. Qiliqiangxin (QLQX) is a compound preparation of 11 Chinese herbal medicines [17], it was approved by the China Food and Drug Administration in 2004 for the treatment of heart failure and was recommended as an ancillary drug in Chinese guidelines for diagnosis and treatment of heart failure. QLQX has been used in China for years to treat heart failure after MI. But there is a lack of scientific explanation on the mechanism of its effect. It has been reported QLQX could improves cardiac function and attenuates cardiac remodeling in rats with experimental myocardial infarction [18] .Recent studies have demonstrated the beneficial effects of QLQX on chronic heart failure [19], Shen S′ study also indicated that QLQX attenuates adverse cardiac remodeling after MI in ovariectomized mice via activation of PPARγ [20]. QLQX can also inhibit cardiomyocyte apoptosis on H9c2 cardiomyocytes [21].. Tao L also think QLQX warrants further investigation as a therapeutic intervention to mitigate remodeling and heart failure after AMI in mice [17]. Due to QLQX’s complex composition, pharmacodynamic pathway and multiple targets, we need more experimental results in vitro and vivo to reveal the mechanism of QLQX to treat MI. In this study, a post-myocardial infarction heart failure model was used to evaluate the effect of QLQX on cardiac function and to explore the protective effect of mitosis on QLQX in cardiac remodeling.

## Methods

### Chemicals

QLQX was provided by Shijiazhuang Yiling Pharmaceutic (Hebei, China). The specific components of QLQX include *Panax ginseng C. A. Mey. Astragalus membranaceus (Fisch.) Bunge. Aconitum carmichaeli Debx. Salvia miltiorrhiza Bge Descurainia sophia(L.)Webb. ex Prantl. Alisma orientalis (Sam.) Juzep. Cinnamomum cassia Presl Carthamus tinctorius L. Periploca sepium Bge. Citrus reticulata Blanco and Polygonatum odoratum (Mill.)Druce* [22] and the labeled compounds have been certified and standardized according to the Chinese Pharmacopoeia (2010). In the current study, QLQX powder was prepared into 25 mg/ml solution with normal saline.

### Surgical operation

Male FVB/NJ mice, aged 8 to10 weeks, were housed and used for experiments. All mice were purchased at the Institute of Biomedical Sciences of Nanjing University (Nanjing, China). Animal breeding, handling and surgical protocols were reviewed, approved and monitored by the Animal Care and Use Committee of Hubei University of Medicine. Briefly, after animals were induced deep anesthesia with 2% isoflurane, the heart was exposed by a left thoracotomy at the third intercostal space, and the anterior descending branch of the left coronary artery (LAD) is ligated with 6–0 suture at a position 2–3 mm below the left atrium. Then, the heart was placed back in situ, followed by evacuating of the air out of the thoracic cavity and closing the skin incision with a 4–0 nylon suture. The mice were placed on an electric blanket to recover and were closely observed. To relieve the pain in mice, one dose of buprenorphine (0.1 mg/kg) was given within 6 h after the operation, and another dose was given the next morning. For experiments, mice were intragastrically treated with QLQX (0.25 g/kg/d, QLQX group) or saline (saline group) for 4 weeks. Animals in the sham group were performed the same operation as the infarction group without ligation of the LAD.

### Echocardiography of heart

The mice were anesthetized using 1.5% isoflurane and the Echocardiography was recorded with a Vevo1100 imaging system using a MS400 transducer. M-mode analysis was used to calculate ejection fraction, ventricle wall thickness, fractional shortening, and intra-ventricle diameter.

### Euthanasia

Intraperitoneal injection of three times the anesthetic dose of 1% sodium pentobarbital solution.

### Immunohistochemistry analysis

Immunohistochemistry was carried out as follows. In short, paraffin-embedded sections are stripped of paraffin, hydrated, and the antigen was repaired by microwave, then incubated in 3% hydrogen peroxide for 10 min to remove endogenous peroxidase, and further incubated with goat serum for 10 min for blocking. Then, the tissue sections were incubated with Pink1 rabbit polyclonal antibody (Proteintech, 23,274–1-AP, 1:200) and Parkin rabbit polyclonal antibody (Proteintech, 14,060–1-AP, 1:200) overnight at 4 °C, followed with a horse radish peroxidase (HRP)-labeled goat anti-rabbit secondary antibody (ZSGB-BIO, PV-9001) for 20 min at room temperature. Next, the sections were incubated with 50ul diaminobenzidine (DAB) for 5–8 min and mounted with neutral gum. Using Olympus BX53 microscope to observe the staining and take photos.

### Assessment of myocardial infarct area

Evan’s blue and 2,3,5-triphenyltetrazolium chloride (TTC) double staining method was used to assess the size of myocardial infarction area [23]. About 0.2–0.3 ml of 2% Evan’s Blue dye solution was injected into the coronary arteries to identify non-ischemic areas. When the right side of the heart turned blue, the heart was rapidly peeled off and frozen at − 20 °C for 30 min. Then the heart was cut into 5 pieces with uniform thickness from apex to atrium, and incubated at 37 °C for 15 min in 1% TTC (0.1 mol/L PBS) at 37 °C for 15 min. The infarct area (INF; white) and area at risk (AAR; red and white) of each segment were used to assess the myocardial infarct size.

### Electron microscopy

Electron Microscopy examination was carried out according to the method of He et al. [24]. In short, 28 days after ligation of left anterior descending coronary artery, a small (about 1-mm^3^) piece of tissue was taken out from the peripheral area of heart infarction, and repidly fixed with 2.5% glutaraldehyde for 2–4 h, then fixed with 2% OsO_4_ for 1 h, and embedded in Acetone: 812 (Ladd Research). Then, the slides were double stained with Uranium and lead and observed and photographed with an HT7700 SS/FEI Tecnai G20 (Hitachi Limited).

### ROS and the mitochondrial membrane potential (∆Ψm) detection

H9c2 cardiomyocytes were donated by Dr. He from Hubei university of medicine [17]. Cells were cultured in high glucose dulbecco’s modified eagle medium (DMEM) supplemented with 10% FBS (Gibco, C11995500), 100 IU/mL of penicillin and 100 μg/mL of streptomycin, and incubated in 95% air, 5% CO_2_. The detection of reactive oxygen species (ROS) and ∆Ψm in H9c2 cells was carried out according to the specifications of ROS detection kit (Beyotime, S0033) and mitochondrial membrane potential detection kit (Solarbio, CA1310). For the determination of ROS, 1 × 10^5^/well H9c2 cells were first cultured under normal conditions (control group), O_2_ 1% or O_2_ 1% + QLQX, and then incubated with reactive oxygen species sensitive dye 2′,7′-Dichlorodihydrofluorescein diacetate (DCFH-DA) solution at 37 °C for 20 min. In order to measure ∆Ψm, cells were treated as described above and then incubated with 5,5′,6,6′-Tetrachloro-1,1′,3,3′-tetraethyl-imidacarbocyanine (JC-1) staining solution (5 mg/ml) at 37 °C for 20 min. After staining, cells were washed twice with JC-1 staining buffer and detected by fluorescence microscope (Olympus FV3000RS). Fluorescence is measured at excitation/emission 485/580 nm (red) and then at excitation/emission 485/530 nm (green). The results were analyzed with Image-Pro Plus software.

### Western blot

Tissues or cells were lysed with protein lysate buffer radio immunoprecipitation assay (RIPA) (Beyotime, P00138). Supernatant of lysate was collected, and the protein concentrations were detected by bicinchoninic acid (BCA) method (Beyotime, P0012). The protein was isolated by 10% sodium dodecyl sulfate polyacrylamide gel electrophoresis (SDS-PAGE) and transferred to polyvinylidene fluoride (PVDF) membrane. The membrane was sealed with 5% skim milk solution at room temperature for 30 min and then incubated overnight at 4 °C with corresponding primary antibody, including Bcl-2 rabbit polyclonal antibody (Beyotime, AF0060, 1:500), Bax rabbit monoclonal antibody (Beyotime, AF1270, 1:500), LC3 rabbit polyclonal antibody (protein technology, 18,725–1-AP, 1:500), Pink1 rabbit polyclonal antibody (protein technology, 23,274–1-AP, 1:500), Parkin rabbit polyclonal antibody. The next day, the membrane was washed with tris buffered saline tween (TBST) buffer and incubated with diluted horseradish peroxidase labeled goat anti-rabbit antibody (Beyotime, A0208, 1:1000) and anti-mouse antibody (Beyotime, A0216, 1:1000) at room temperature for 1 h. The ultra-sensitive chemiluminescence kit BeyoECL Star was used for color development, and the results were analyzed with image J 8.0.

### Analysis of mitophagic vacuoles

H9c2 cells were transfected with green fluorescent protein-tagged microtubule—associated protein 1light chain 3 (Ad-GFP-LC3) and red fluorescent protein -tagged HBmTur-Mito and Ad-HBm (Tur-Mito-RFP, HANBIO), according to the manufacturer’s instructions. The next day, cells treated with or without 1% O_2_ or 1% O_2_ + QLQX for 12 h. After the treatments, the cells were inspected with a fluorescence microscope (Olympus FV3000RS). The mitophagy was quantified by calculating the percentage of GFP-LC3 and Mito-RFP copositive autophagic vacuoles in cells.

### TUNEL assays

Tissue slides and cells which were cultured with 1% O2 or 1% O2 + QLQX for 12 h, were reacted with terminal deoxynucleotidyl transferase (TdT) enzyme and 2′-Deoxyuridine 5′-Triphosphate (dUTP) at 37 °C for 1 h. Then 4′, 6- diamino − 2- phenylindole (DAPI) (Beyotime, C1006) was used to stain the nucleus for 5 min. Apoptosis was observed and photographed by fluorescence microscope (Olympus FV3000RS).

### Statistical analysis

Prism 7.0 (Graph Pad) was used for statistical analysis of the data. All data were expressed as means ±standard deviation (SD). The comparison of measurement data between two groups was carried out by independent-sample T test. Survival curves were analyzed with the Kaplan-Meier tests. A *p* value less than 0.05 was considered statistically significant.

## Results

### QLQX improves survival rate and cardiac function after MI

**Fig. 1:**
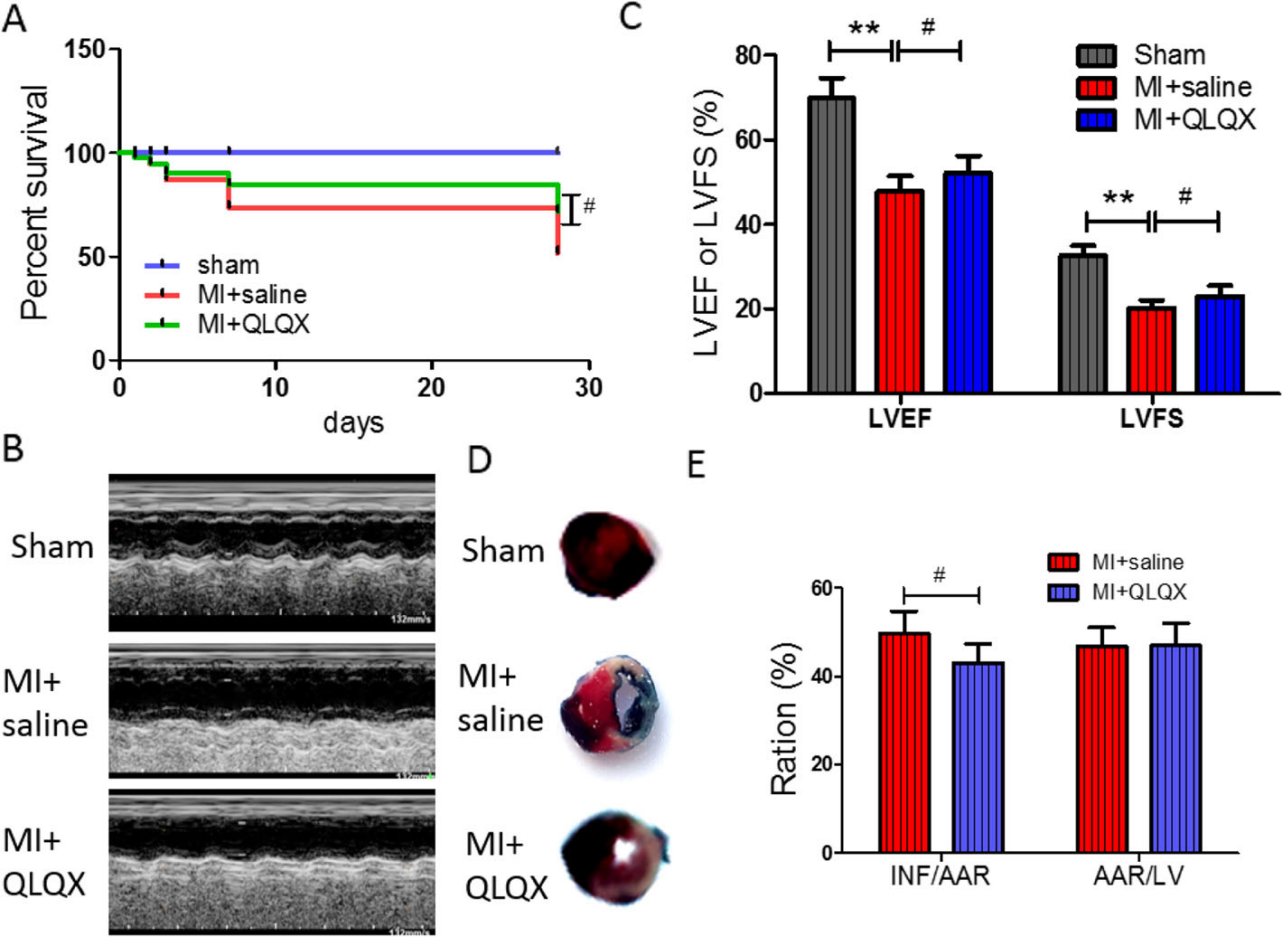
Qiliqiangxin (QLQX) improves survival rate and cardiac function after myocardial infarction (MI). **a**: Survival rate. FVB/NJ mice were subjected to sham or MI operation. The number of dead mice over a 4-wk period was counted daily. Survival curves were analyzed with the Kaplan-Meier tests. Data are presented as mean ± SD, ^#^compared to saline group, *P* < 0.05, *n* = 10 per group. **b**: Echocardiogram analysis. Representative echocardiographic images of FVB/NJ mice 4 weeks after MI surgery. **c**: QLQX improves cardiac function including preserving left ventricular fractional shortening (LVFS) and left ventricular ejection fraction (LVEF). Data are presented as mean ± SD, ^**^compared to Sham group, *P* < 0.01, ^#^ compared to saline group, *P* < 0.05, *n* = 8 per group. **d**: Evan’s blue/TTC stain. Heart tissue sections were stained with Evan’s blue/TTC at 4 weeks after MI surgery. The infarct area (INF: white) and area at risk (AAR: red); blue is the normal myocardial blood supply area. **e**: The ratio of INF/AAR and AAR/LV. Data are presented as mean ± SD, ^#^ compared to saline group, P < 0.05, *n* = 6 per group. The comparison of measurement data between two groups was carried out by independent-sample T test

To investigate the effect of QLQX on MI, the survival rate of mice after MI operation was observed. Survival rates of saline group and QLQX-treated group were similar within the first 24 h. Nevertheless, the mortality rate of saline group mortality was higher than QLQX group over 28 days (37.5% for saline vs 22.5% for QLQX; *P* < 0.05, Fig. 1a).

Echocardiogram was used to assess cardiac function of each group. Compared to sham controls, the contractile function of the infarcted anterior wall in both saline and QLQX groups were impaired from the 2-D M-mode imaging of the left ventricle. Furthermore, the cardiac function in the saline group was significantly worse than that in QLQX group (Fig. 1b). QLQX consistently improved cardiac functions including left ventricular ejection fraction (LVEF) and left ventricular shortening fraction (LVFS) (Fig. 1c, *P* < 0.05). LVEF values were 69.87 ± 4.68%, 47.84 ± 3.56% and 52.16 ± 4.15%, and LVFS values were 32.62 ± 2.33%, 20.22 ± 1.8% and 22.92 ± 2.56% for sham, saline and QLQX groups respectively.

The myocardial infarction size was measured by evan’s blue/TTC double stain to determine the effects of QLQX on myocardial infarction. Representative myocardial infarct size images were shown in Fig. 1d. After 28 days of MI, the infarct size of the saline group and the QLQX group increased significantly. Compared with saline group, the infarct area in QLQX group was significantly reduced (INF/AAR: 49.68 ± 5.13% vs. 43.08 ± 4.23%, *P* < 0.05, respectively. Figure 1e). There was no significant difference between saline group and QLQX group in AAR/LV (AAR /LV: 46.78 ± 24.34% vs. 47.13 ± 5.01%, respectively. *P* > 0.05, Fig. 1e).

### QLQX reduced cell apoptosis after myocardial infarction (MI) during remodeling phase

**Fig. 2:**
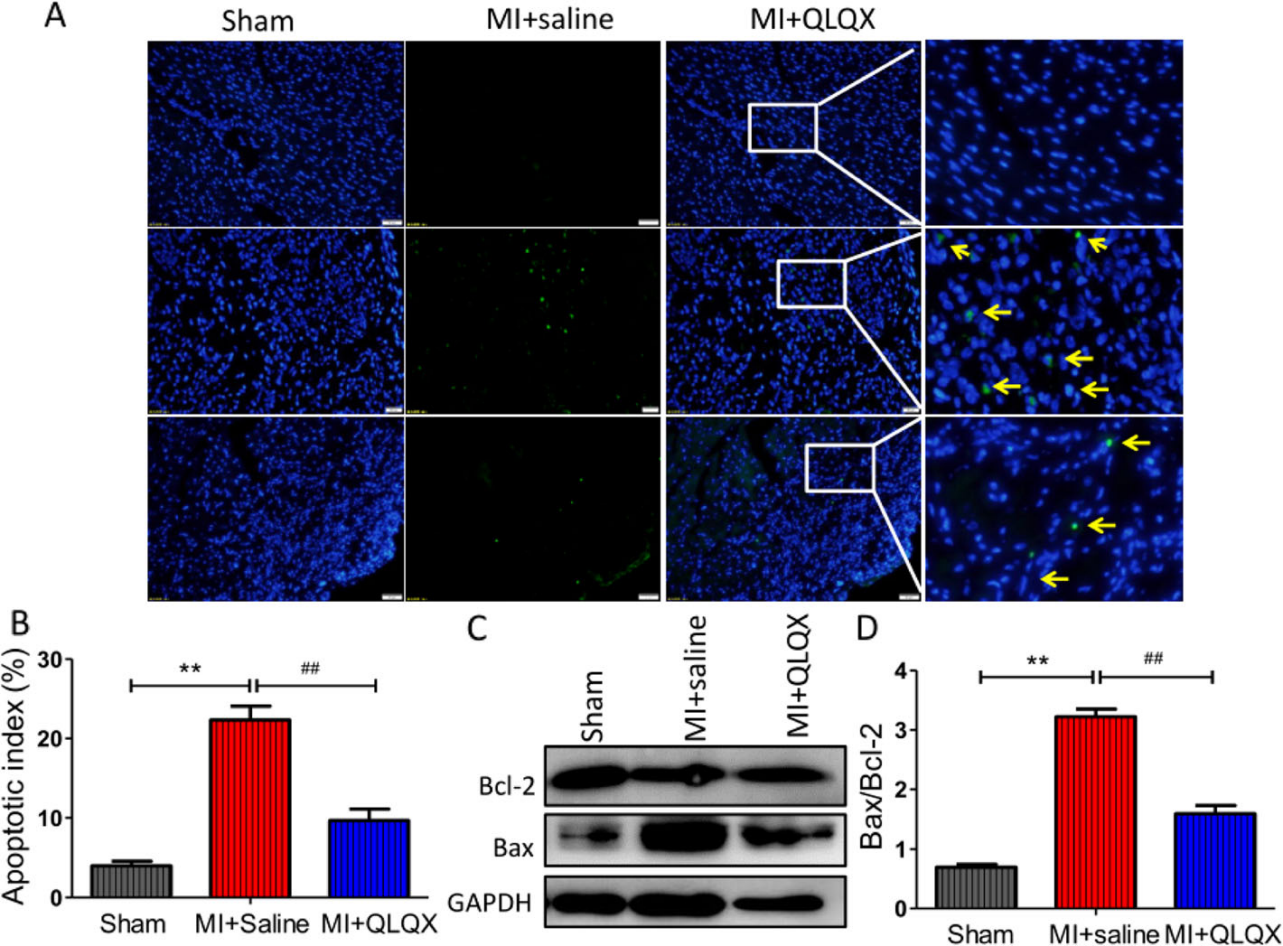
Qiliqiangxin (QLQX) reduces cardiomyocyte apoptosis after myocardial infarction (MI) during remodeling phase. **a**: TUNEL Assays detected the apoptosis cells in the infarction peripheral tissues. The yellow arrow indicated the apoptosis cells. **b**: Column diagram showed the percentage of apoptosis cells, data are presented as mean ± SD, ^**^ Compared to sham group, *P* < 0.01; ^##^ Compared to saline group, *P* < 0.01, *n* = 4 per group. **c**: Western blot detected the apoptosis marker Bax and Bcl-2. **d**: Column diagram showed the ratio of Bax/Bcl-2. Data are presented as mean ± SD, ^**^ Compared to sham group, *P* < 0.01; ^##^ Compared to saline group, *P* < 0.01, *n* = 4 per group. The comparison of measurement data between two groups was carried out by independent-sample T test

Myocardial apoptosis is another characteristic of ventricular remodeling after myocardial infarction. The apoptotic cells were significantly decreased in the QLQX group as assessed by TUNEL staining when compared to saline group. The percentages of apoptotic cells were 4.03 ± 0.58, 22.33 ± 1.76 and 9.67 ± 1.45 for sham, saline and QLQX groups respectively (Fig. 2a&B, *P* < 0.01). The expression levels of Bax and Bcl-2 were detected by Western blot (Fig. 2c). The ratio of Bax/Bcl-2 was used to measure the apoptosis of cardiomyocytes and it was significantly increased after MI. However, QLQX could obviously reverse this phenomenon (Fig. 2d). The ratios of Bax/Bcl-2 in sham, saline and QLQX groups were 0.7 ± 0.05, 3.22 ± 0.13 and 1.58 ± 0.14 respectively (*P* < 0.01).

### QLQX increased mitophagy after MI surgery

**Fig. 3:**
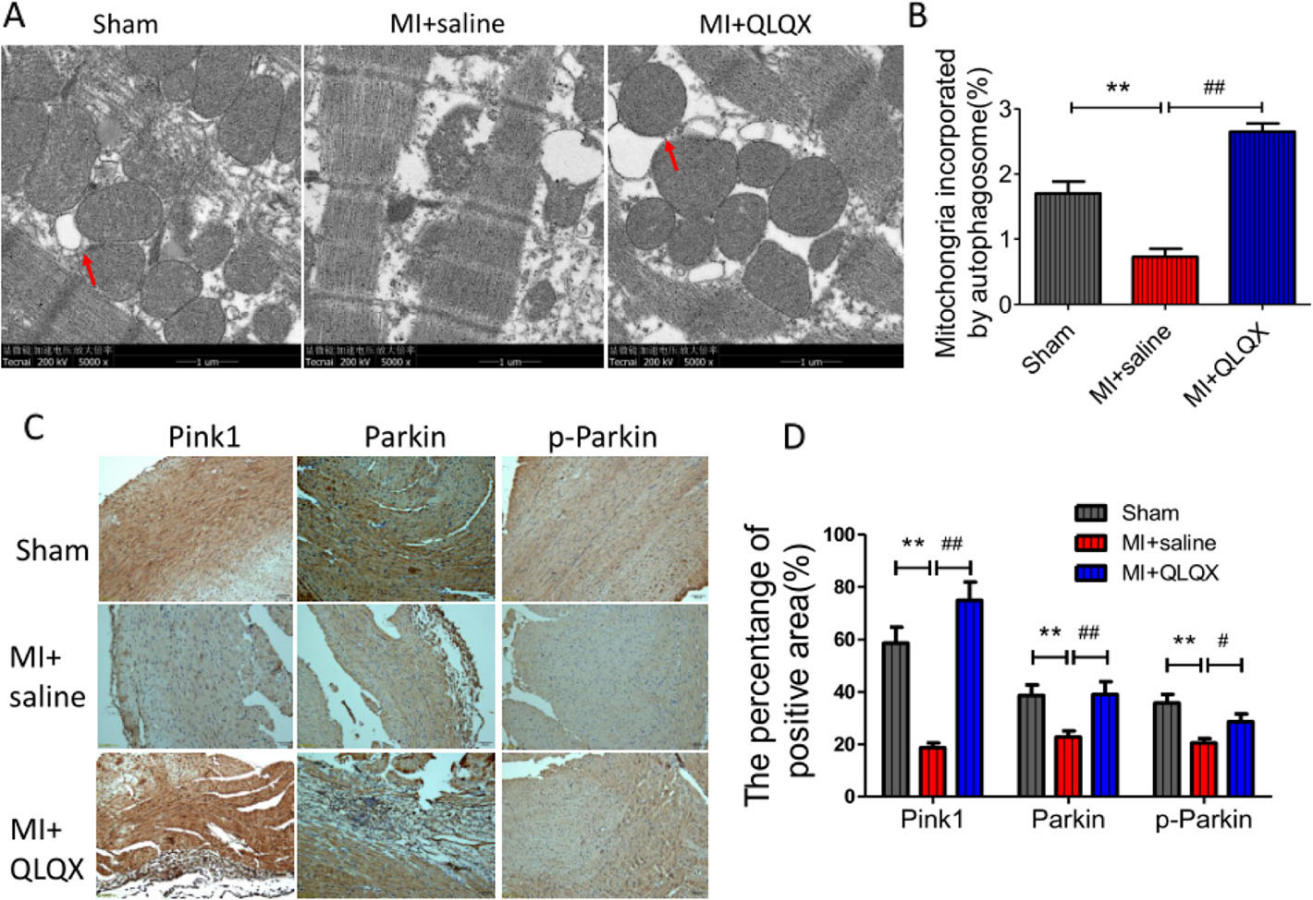
QLQX increases mitophagy after MI surgery. **a**: Transmission electron microscopy (5000×) detected mitochondrial structure and autophagy in infarction peripheral tissues, the red arrows indicated the autophagosome. **b**: Column diagram showed the percentage of mitochongria incorporated by autophagosome, data are presented as mean ± SD, ^**^ Compared to sham group, *P* < 0.01; ^##^ Compared to saline group, *P* < 0.01, *n* = 3 per group. **c**&**d**: Immunochemical staining detected the expression of Pink1, Parkin and p-Parkin in the heart at 4 weeks after MI. Representative images were selected from 3 separate experiments. Data are presented as mean ± SD, ^**^ Compared to sham group, *P* < 0.01; ^##^ Compared to saline group, *P* < 0.01, ^#^ Compared to saline group, *P* < 0.05, *n* = 4 per group. The comparison of measurement data between two groups was carried out by independent-sample T test

To further detect the underline mechanism, transmission electron microscopy (TEM) was used to observe the mitochondrial structure. The results showed that on day 28 the mitochondrial structure was preserved and orderly arranged, and there were no significant structural variations in sham group. In saline group, mitochondria were aggregated at the myofilament breaks, and some of them were evidently vacuolated. The mitochondria of QLQX group were arranged more regularly than that of saline group, and some of them were filled in the area of myofibril breakage and loss (Fig. 3a). The percentage of mitochongria incorporated by autophagosome of QLQX group was significantly higher than the saline group (Fig. 3b). Further study indicated that QLQX treatment could repress the decrease of Pink1, Parkin and pParkin induced by MI, which was demonstrated in the immunohistochemistry analysis (Fig. 3c). The positive area of Pink1, Parkin and pParkin in saline group was significantly decreased when compared to sham group, but QLQX treatment could suppress this phenomenon. The Pink1 positive areas were 58.67 ± 5.84, 18.68 ± 1.8 and 74.87 ± 7.03 in sham, saline and QLQX groups respectively (Fig. 3d, *P* < 0.01)., the Parkin positive areas were 38.67 ± 4.03, 22.78 ± 2.34 and 39.13 ± 4.18 in sham, saline and QLQX groups respectively (Fig. 3d, *P* < 0.01), the pParkin positive areas were 35.78 ± 3.35, 20.56 ± 1.63 and 28.65 ± 2.97 in sham, saline and QLQX groups respectively (Fig. 3d, *P* < 0.05).

### Effect of QLQX on hypoxic injury in H9c2 cardiac cells

**Fig. 4:**
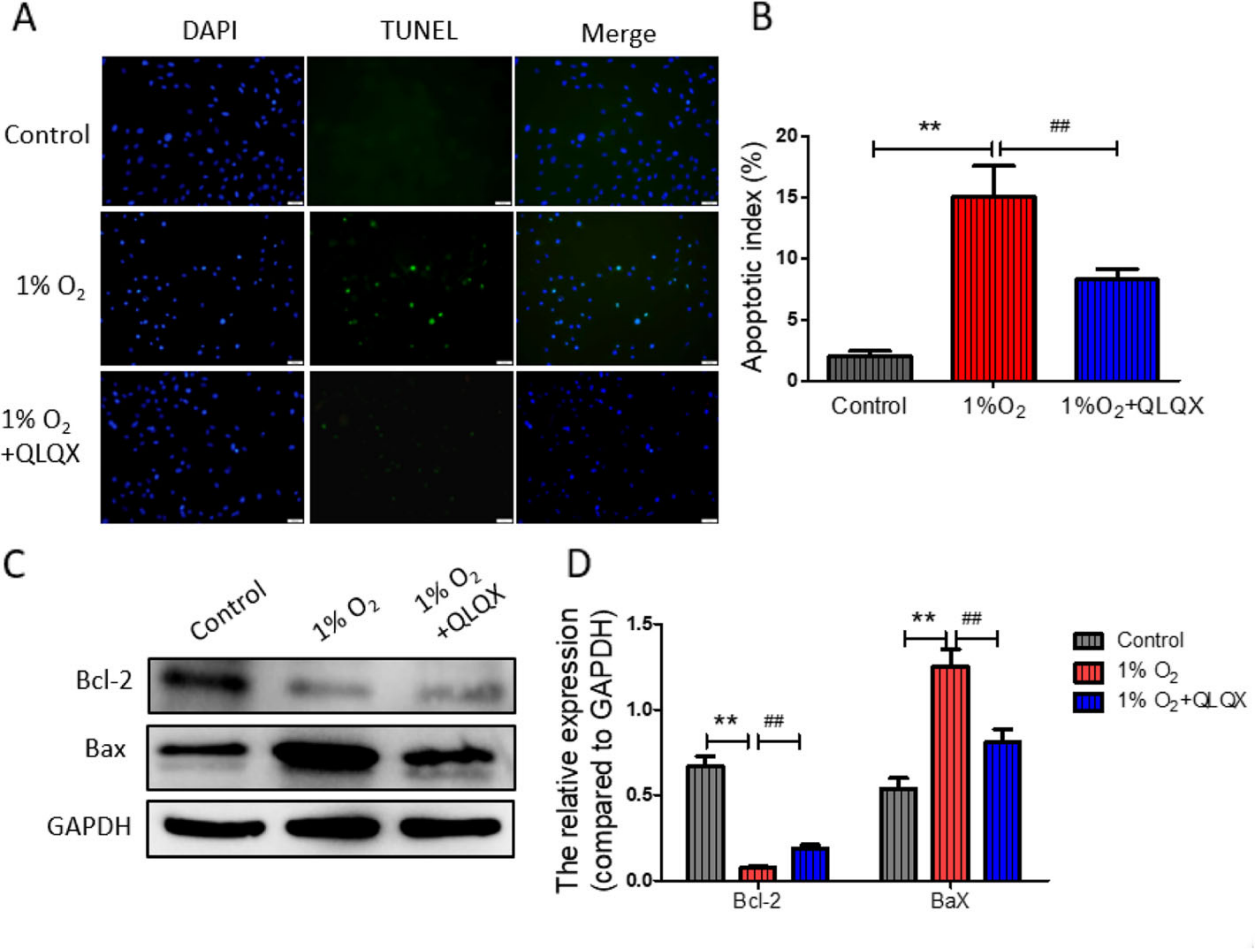
Effect of QLQX on hypoxic injury in H9c2 cardiac cells. **a**: TUNEL staining was used to detect the apoptosis of H9c2 cells treated with 1% O_2_ or 1% O_2_ + QLQX for 12 h, the green fluorescence indicated the apoptotic cells. **b**: Column diagram showed percentage of apoptosis cells. Data are presented as mean ± SD, ^**^ Compared to control group, *P* < 0.01; ^##^ Compared to 1% O_2_ group, *P* < 0.01. *n* = 3 per group. **c**: Western blot analysis of Bax and Bcl-2 levels in H9c2 cells. **d**: Column diagram showed the relative expression levels of Bax and Bcl-2. The statistical data are presented as mean ± SD of three independent experiments. Data are presented as mean ± SD, ^**^ Compared to control group, *P* < 0.01; ^##^ Compared to 1% O_2_ group, *P* < 0.01. *n* = 3 per group. The comparison of measurement data between two groups was carried out by independent-sample T test

We assessed the apoptosis in different group with TUNEL assay. The viability of H9c2 cardiac cells following hypoxia treatment was shown in Fig. 4a. The percentage of apoptosis cell after hypoxia treatment was markedly increased (2.07 ± 0.41% vs. 15.36 ± 2.8% for control vs. 1%O_2_, *P* < 0.01). This effect was significantly alleviated by QLQX treatment (QLQX: 8.25 ± 0.87 vs.15.36 ± 1.8% for 1%O_2_ + QLQX vs. 1%O_2_, *P* < 0.01, Fig. 4 b). The results of western blot revealed that compared to saline group, the expression of pro-apoptotic molecule Bax was decreased, while the anti-apoptotic molecule B-cell lymphoma 2 (Bcl-2) was increased in QLQX-treated mice after MI (Fig. 4c&d). Collectively, these datas confirmed that QLQX attenuates hypoxia-induced H9c2 cardiomyocyte apoptosis.

### QLQX reduced ROS production and increased ∆Ψm

**Fig. 5:**
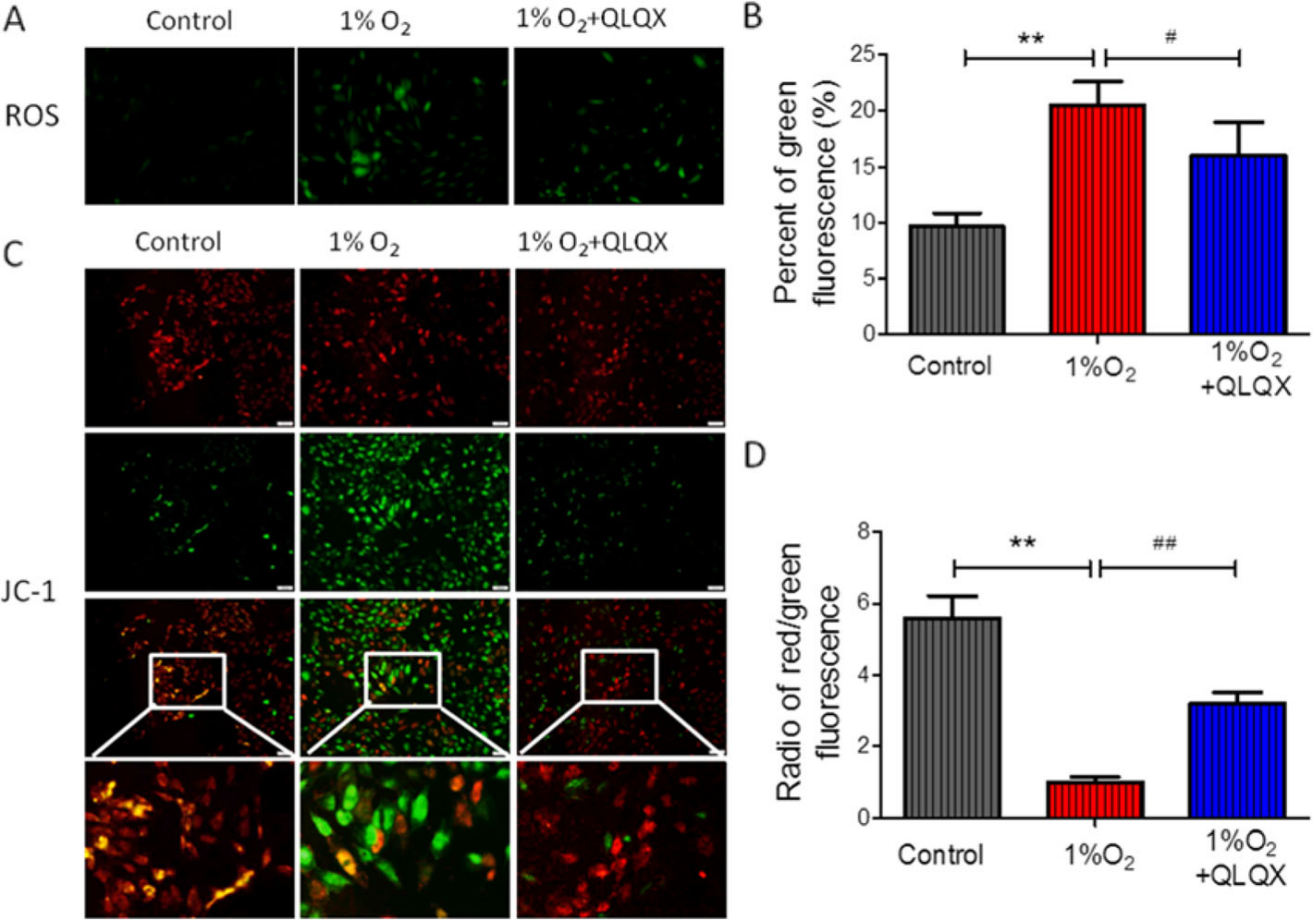
QLQX reduced ROS production and increased ∆Ψm. **a**: Representative images of ROS staining. **b**: Quantifications of ROS fluorescence intensity in different groups. Data are presented as mean ± SD, ^**^ Compared to control group, *P* < 0.01; ^##^ Compared to 1% O_2_ group, *P* < 0.01. *n* = 3 per group. **c**: Mitochondrial membrane potential (∆Ψm) detected by JC-1 staining. Red fluorescence represents the mitochondrial aggregate form of JC-1, indicating an intact mitochondrial membrane potential. Green fluorescence represents the monomeric form of JC-1, indicating the dissipation of ∆Ψm. **d**: Ratio of the red/green fluorescence intensity. Data are presented as mean ± SD, ^**^ Compared to control group, *P* < 0.01; ^##^ Compared to 1%O_2_ group, *P* < 0.01. *n* = 3 per group. The comparison of measurement data between two groups was carried out by independent-sample T test

The change of ROS generation was detected by using the DCFH-DA dye. Hypoxia would increase the staining of DCFH-DA, whereas the QLQX treatment could reduce the staining of DCFH-DA induced by hypoxia (Fig. 5a). The differences of ROS production between the three groups were statistically significant. Compared to control group (9.67 ± 1.2), the percentage of green fluorescence positive cells in hypoxia group significantly increased, which was 20.5 ± 2.1 (*P* < 0.01). However, QLQX treatment clearly alleviated this hypoxia-induced cell apoptosis (16 ± 3.1, *P* < 0.01, Fig. 5b). To investigate the mitochondrial function, the mitochondrial potential (∆Ψm) was measured and showed a dramatical decrease in hypoxia group (Fig. 5c). However, QLQX treatment restored the ∆Ψm, the ratios of red to green fluorescence positive cells were 5.6 ± 0.61, 1 ± 0.15 and 3.2 ± 0.3 for control, 1% O_2_ and 1% O_2_ + QLQX group respectively (*P* < 0.01, Fig. 5d).

### QLQX increased mitophagy level in hypoxic injured H9c2 cells

**Fig. 6:**
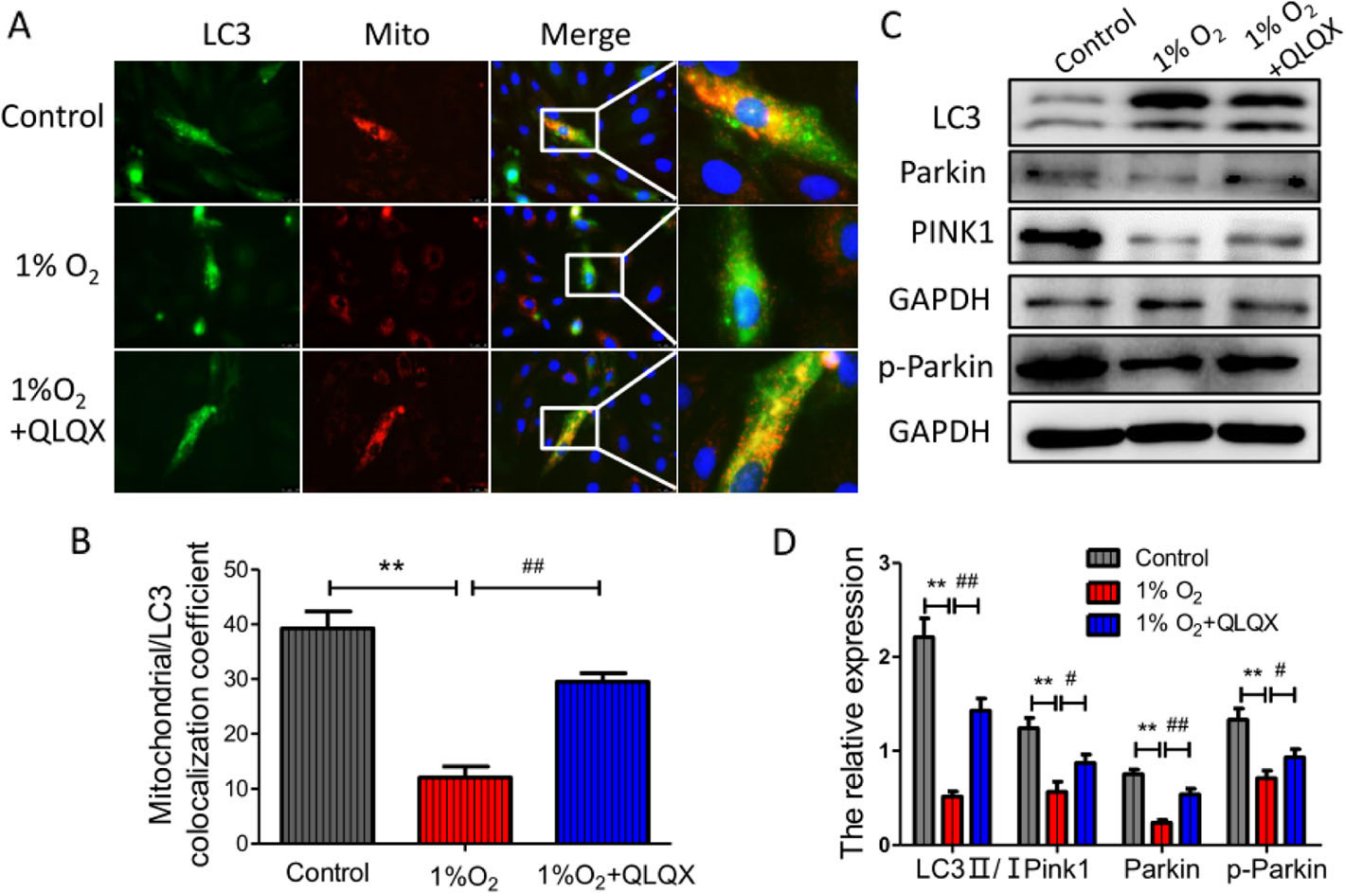
QLQX increased mitophagy level in hypoxic injured H9c2 cells. **a**: Representative photomicrographs of GFP-LC3 and red fluorescent protein (RFP)-tagged HBmTur-Mito staining in H9c2 cells. The green fluorescence dots indicate the autophagy. The red fluorescence dots indicate the mitochondria. The orange fluorescence indicates the mitophagy, 400×. **b**: The co-localization efficient of Mitochondria and LC3. Data are presented as mean ± SD, ^**^ Compared to control group, *P* < 0.01; ^##^ Compared to 1% O_2_ group, *P* < 0.01. *n* = 3 per group. **c**: Western blot analysis of mitophagy markers of LC3, Parkin, Pink1and p-Parkin in different groups. **d**: Column diagram showing the relative expression level of LC3, Pink1,Parkin and pParkin. Data are presented as mean ± SD, ^**^ Compared to control group, *P* < 0.01; ^##^ Compared to 1% O_2_ group, *P* < 0.01, ^#^ Compared to 1% O_2_ group, *P* < 0.05, *n* = 3 per group. The comparison of measurement data between two groups was carried out by independent-sample T test

To detect the influence of QLQX on the process of mitophagy in hypoxic injured H9c2 cells, we transfected H9c2 cells with adenovirus that stably expresses the GFP-tagged LC3 and RFP-tagged HBmTur-Mito, which can co-localize autophagosome and mitochondria. As shown in Fig. 6a, compared to control group, the number of green fluorescent protein (GFP) and red fluorescent protein (RFP) co-positive dots in 1% O_2_ group were significantly decreased. However, when treated with QLQX, the number of autophagosome was increased. The mitochondrial/LC3 co-localization coefficient in control, 1% O_2_ and QLQX groups were 39.25 ± 3.19, 12 ± 2.04, 29.5 ± 1.56 respectively (Fig. 6b, *P* < 0.05). Compared to saline group, western blot results also indicated that the level of Pink1, Parkin and pParkin were increased in the QLQX treatment group (Fig. 6c&d). These results showed that QLQX treatment could increase the mitophagy level in H9c2 cells.

## Discussion

Myocardial infarction (MI) is an irreversible death of myocardial cells in the corresponding area of coronary artery occlusion. The high incidence and younger age of myocardial infarction have become more and more severe. The World Health Organization (WHO) estimates that about 23 million people accounting for 12.8% of total deaths will die of cardiovascular disease each year by 2030 [25]. The damage of heart function after myocardial infarction and the consequent decline in quality of life have also become increasingly serious problems in public health care [26]. Therefore, how to protect myocardium and improve cardiac function as much as possible after myocardial infarction is an urgent challenge for clinicians.

Cardiomyocyte apoptosis is vital to the pathological process of heart failure. Cardiomyocytes lost through apoptosis are not renewable, thus inhibiting cardiomyocyte apoptosis may have great influence to the therapeutic of heart failure. QLQX is a Chinese patent medicine developed according to the theory of collateral disease. It has exhibited remarkable curative effect in clinical applications and holds the traditional functions of strengthening heart, diuresis and vasodilation [17, 19, 20, 24]. Basic research shows that QLQX could restrain hypoxia-induced injury in primary rat cardiac microvascular endothelial cells via promoting glycolysis in a HIF-1a-dependent manner [27]. During hypoxia, cardiac microvascular endothelial cells (CMVECs) promote the expression of vascular endothelial growth factor (VEGF) and QLQX could attenuate this injury via facilitating hypoxia inducible factor-1α(HIF-1α)-dependent glycolysis [28]. However, the mechanism of QLQX on apoptosis still remains unknown.

In the present study, we established mice myocardial infarction model by ligation of left coronary artery [29]. The results showed that QLQX could significantly reduce the infarct area, improve the heart function and survival rate of mice at 28 days after surgery. Bcl-2-related protein family is the main regulator of cell apoptosis signal transduction pathway, and the ratio of anti-apoptosis protein Bcl-2 to pro-apoptosis protein Bax determines whether cells undergo apoptosis when stimulated by apoptosis signal [28, 30]. Our study found that after ligation of left coronary artery, the expression of Bax increased significantly while the expression of Bcl-2 decreased significantly in MI + saline group compare to sham group. However, QLQX could obviously restore the imbalance of Bcl-2/Bax. This result indicated that QLQX plays an anti-apoptosis protective role on myocardial cells. Apoptosis is a highly programmed cell death. Cardiomyocyte apoptosis contains cell surface death receptor signaling pathway and intracellular mitochondrial pathway two classical signaling pathways. The cell surface death receptor signaling pathway is mainly stimulated by Fas ligand or ligand of death receptor, such as tumor necrosis factor α (TNG-α), while mitochondrial pathway can be activated by myocardial hypoxia, ischemia, oxidative stress, anticancer drugs, deoxyribonucleic acid (DNA) damage and other stimuli [31].

Mitochondria are important organelles widely found in eukaryotic cells. It is the main site for cells to oxidize and produce adenosine-triphosphate (ATP), providing 95% of the energy needed for cell life activities, known as the “cell power plant”. The structure and biochemical functions of mitochondria are complex, sensitive and changeable. Changes of intracellular and extracellular factors can cause structural abnormality and malfunction of mitochondria [32, 33]. Therefore, mitochondria are often used as indicators for the changes of microenvironmental factors and even for the diagnosis of certain disease [34]. Mitophagy, as a selective autophagy, is a process of removing damaged or excessive mitochondria in cells [35], which refers to the process of depolarization damage of mitochondria in cells under the stimulation of ischemia, hypoxia, oxidative stress and aging, thus activating autophagy-lysosome pathway, completing the degradation of damaged mitochondria and maintaining the homeostasis of cell environment. Mitophagy could timely remove damaged or excessive mitochondria thereby maintaining the normal function of the body cells. In this study, by using transmission electron microscopy, we found that after MI, the autophagy level of left ventricular decreased significantly, while QLQX significantly increased the level of myocardial autophagy after MI. In vitro H9c2 cell assays also showed that QLQX treatment could significantly improve mitochondrial dysfunction caused by hypoxia, presumably through reducing ROS production and the level of mitochondrial membrane potential. QLQX treatment also facilitated the mitochondrial/LC3 co-localization. These results indicated that QLQX could significantly improve myocardial injury and increase mitophagy level after MI.

Mitophagy includes four stages: earlier stage, early stage, middle stage and late stage. Each stage has different characteristics. The proteins involved in this process mainly includes: protein encoding mitochondrial outer membrane kinase (Pink1), E3 ubiquitin ligase (Parkin), B cell lymphoma/ leukemia-2 (Bcl-2), adenovirus interaction protein 3 (BNIP-3), autophagy-related gene (ATG) protein and Uth1 protein. Pink1 and Parkin proteins can induce mitophagy, remove excessive or damaged mitochondria, and maintain the stability of mitochondrial structure and function. The BNIP3 protein located in the outer membrane of mitochondria plays a significant role in the clearance of mitophagy in the process of mitochondrial development. ATG protein system is mainly involved in the formation of early mitophagy. Uth1 protein is a protein located in mitochondria. Mutation or deletion of Uthl will cause cells to be unable to remove damaged or aging mitochondria [35–37]. In our experiment, the data showed that Pink1, Parkin and pParkin levels decreased after MI, while QLQX could improve this phenomenon. This point was also confirmed in our in vitro cell experiments.

## Conclusions

Taken together, our results indicated for the first time that QLQX could obviously improve the survival rate and the heart function of mice after MI presumably through Pink 1/Parkin signaling pathway-mediated increase of mitophagy and could reduce cardiomyocytes apoptosis. This study will provide new supportive evidence for the clinical application of QLQX.

## Supplementary information

**Additional file 1.**

**Additional file 2.**

**Additional file 3.**

## Data Availability

All data is contained within the manuscript and additional files.

## Abbreviations

QLQX: Qiliqiangxin
MI: Myocardial infarction
TUNEL: TdT-mediated dUTP Nick-End Labeling
DMEM: Dulbecco’s modified eagle medium
ROS: Reactive oxygen species
LAD: Left coronary artery
DAB: Diaminobenzidine
HRP: Horse radish peroxidase
TTC: 2,3,5-triphenyltetrazolium chloride
INF: Infarct area
AAR: Area at risk
DCFH-DA: 2′,7′-Dichlorodihydrofluorescein diacetate
JC-1: 5,5′,6,6′-Tetrachloro-1,1′,3,3′-tetraethyl-imidacarbocyanine
RIPA: Radio immunoprecipitation assay
BCA: Bicinchoninic acid
SDS-PAGE: Sodium dodecyl sulfate polyacrylamide gel electrophoresis
PVDF: Polyvinylidene fluoride
TBST: Tris buffered saline tween
Ad-GFP-LC3: Green fluorescent protein-tagged microtubule—associated protein 1light chain 3
Tur-Mito-RFP, HANBIO: Red fluorescent protein -tagged HBmTur-Mito and Ad-HBm
TdT: Terminal deoxynucleotidyl transferase
DAPI: 4′, 6- diamino − 2- phenylindole
dUTP: 2′-Deoxyuridine 5′-Triphosphate
SD: Standard deviation
LVEF: Left ventricular ejection fraction
LVFS: Left ventricular shortening fraction
TEM: Transmission electron microscopy
GFP: Green fluorescent protein
RFP: Red fluorescent protein
WHO: World Health Organization
CMVECs: Cardiac microvascular endothelial cells
VEGF: Vascular endothelial growth factor
HIF-1α: Hypoxia inducible factor-1α
TNG-α: Tumor necrosis factor α
DNA: Deoxyribonucleic acid
ATP: Adenosine-triphosphate
ATG: Autophagy-related gene
Pink1: Protein encoding mitochondrial outer membrane kinase
Parkin: E3 ubiquitin ligase
Bcl-2: B cell lymphoma/ leukemia-2
BNIP-3: Adenovirus interaction protein 3
