# Supplementary material for: Qiliqiangxin reduced cardiomyocytes apotosis and improved heart function in infarcted heart through Pink1/Parkin -mediated mitochondrial autophagy
Source: BMC Complement Med Ther. 2020 Jul 2;20:203. doi: 10.1186/s12906-020-02992-7 (PMC7330946; doi:10.1186/s12906-020-02992-7)
Supplement: Supplementary file 1 — Additional file 1. [file 12906_2020_2992_MOESM1_ESM.pptx]

## Slide 1
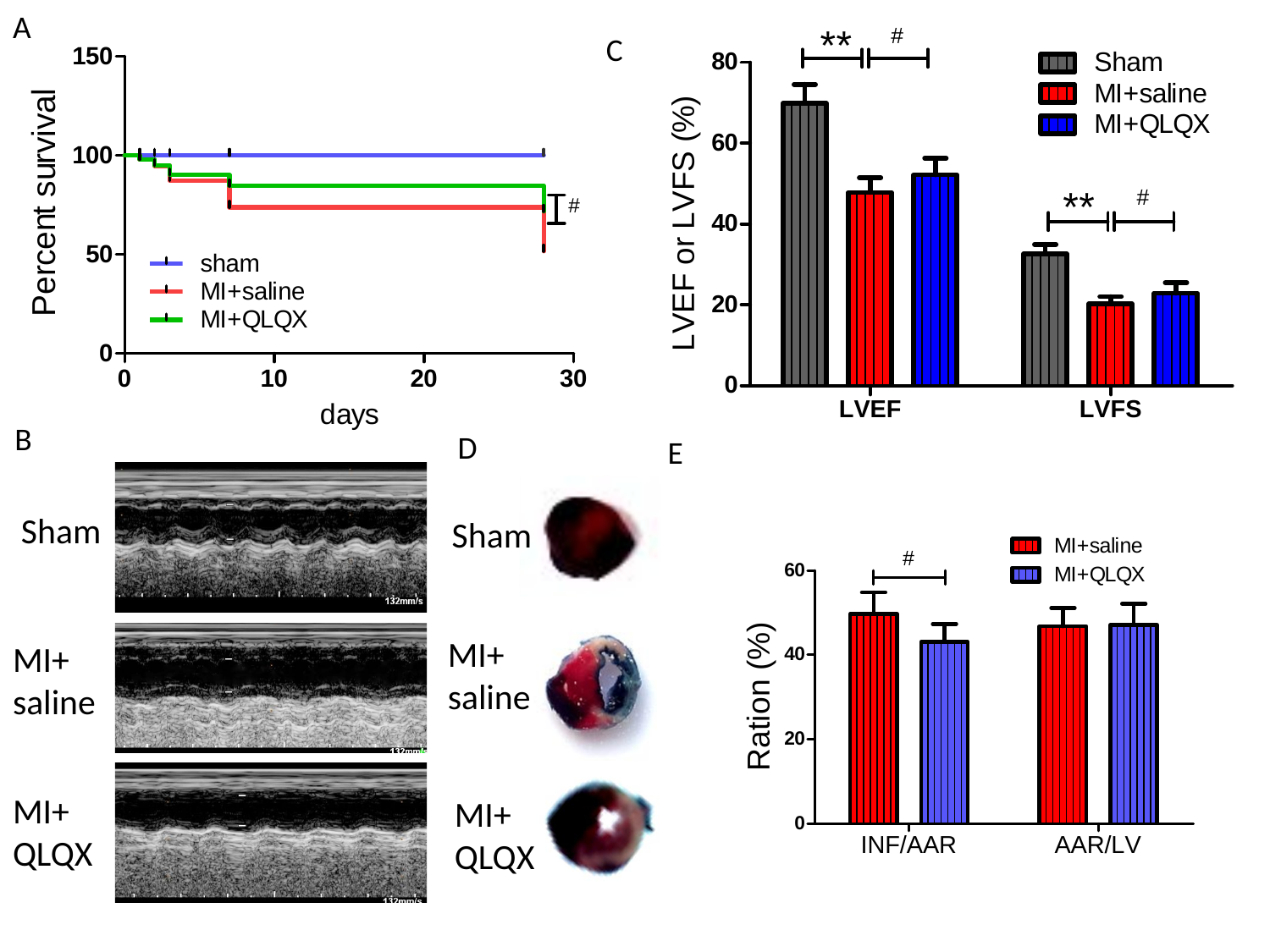

A
C
B
D
E
Sham
MI+
saline
MI+
QLQX
Sham
MI+
saline
MI+
QLQX

## Slide 2
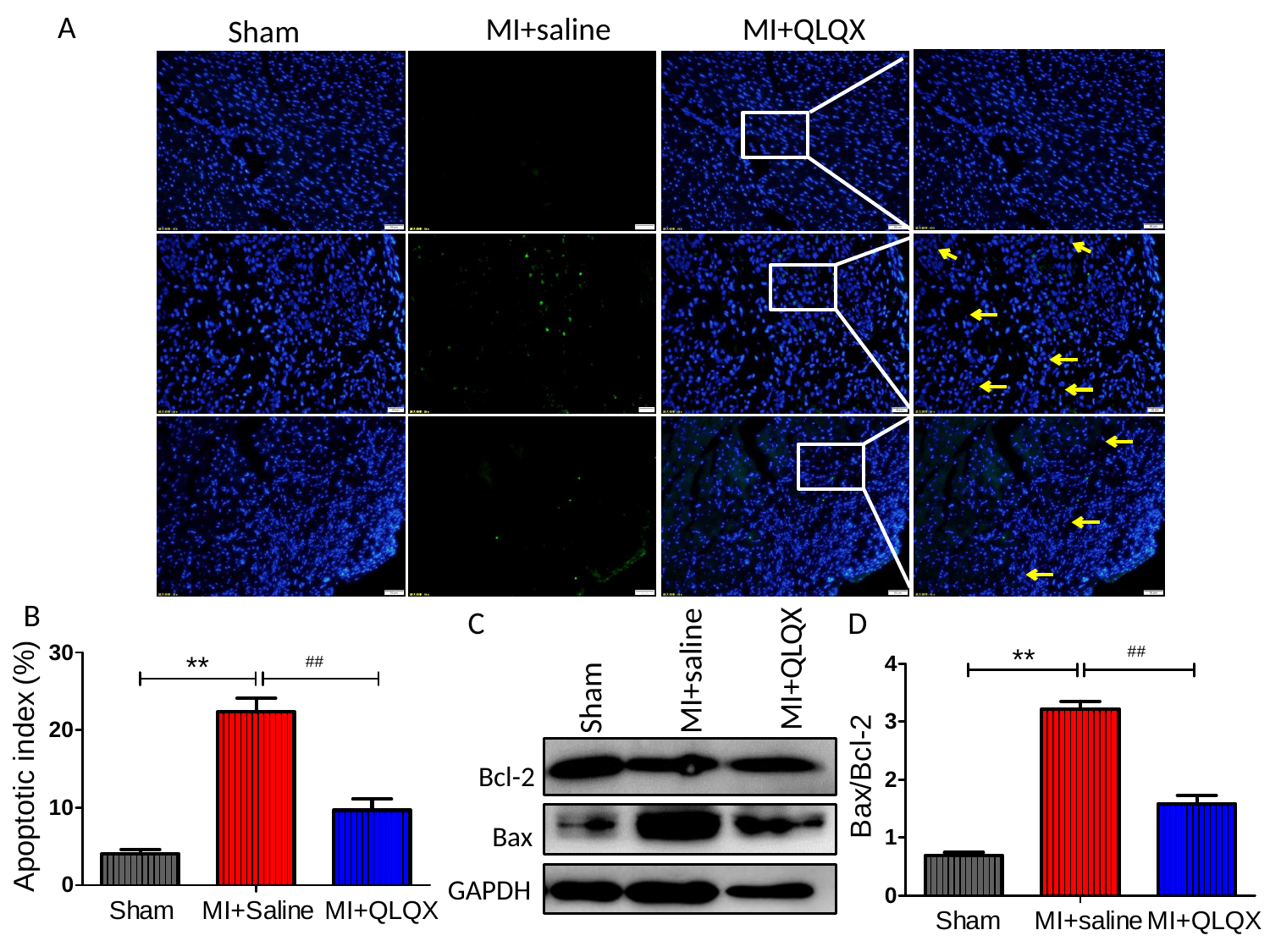

A
MI+saline
MI+QLQX
Sham
B
C
D
MI+QLQX
MI+saline
Sham
Bcl-2
Bax
GAPDH

## Slide 3
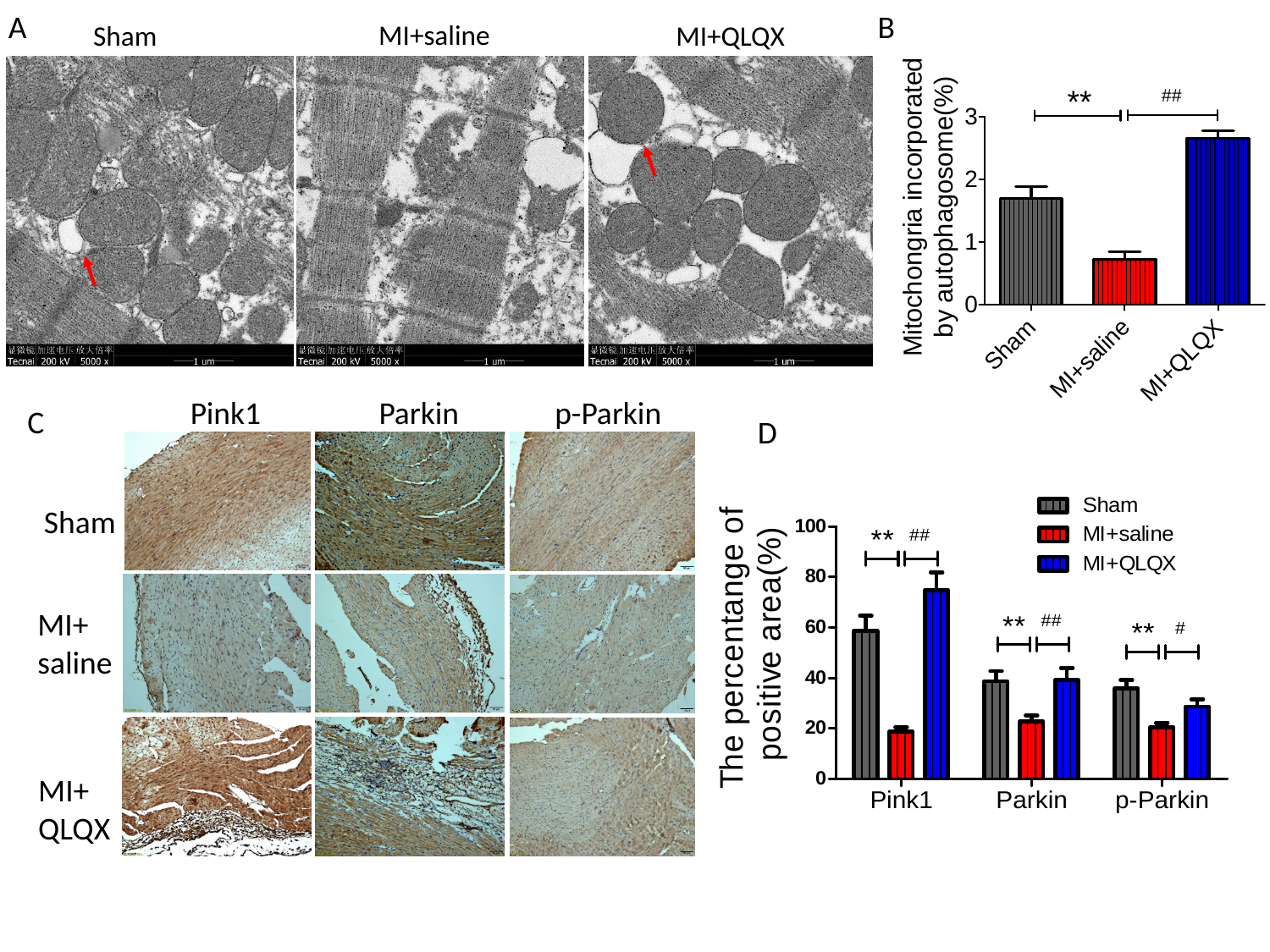

A
B
MI+saline
Sham
MI+QLQX
Pink1
Parkin
p-Parkin
C
Sham
MI+
saline
MI+
QLQX
D

## Slide 4
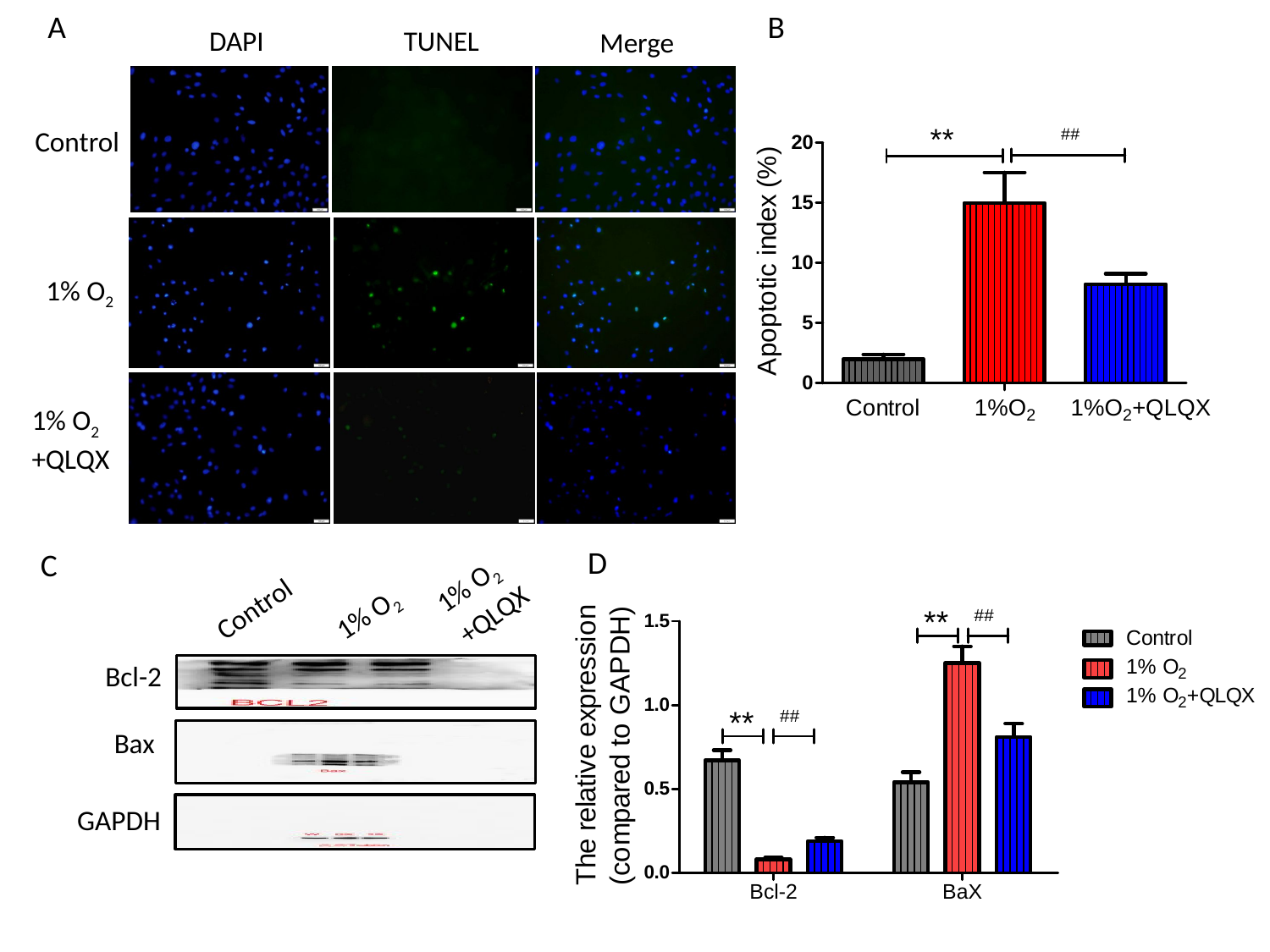

A
B
DAPI
TUNEL
Merge
Control
1% O2
1% O2
+QLQX
D
C
1% O2
+QLQX
1% O2
Control
Bcl-2
Bax
GAPDH

## Slide 5
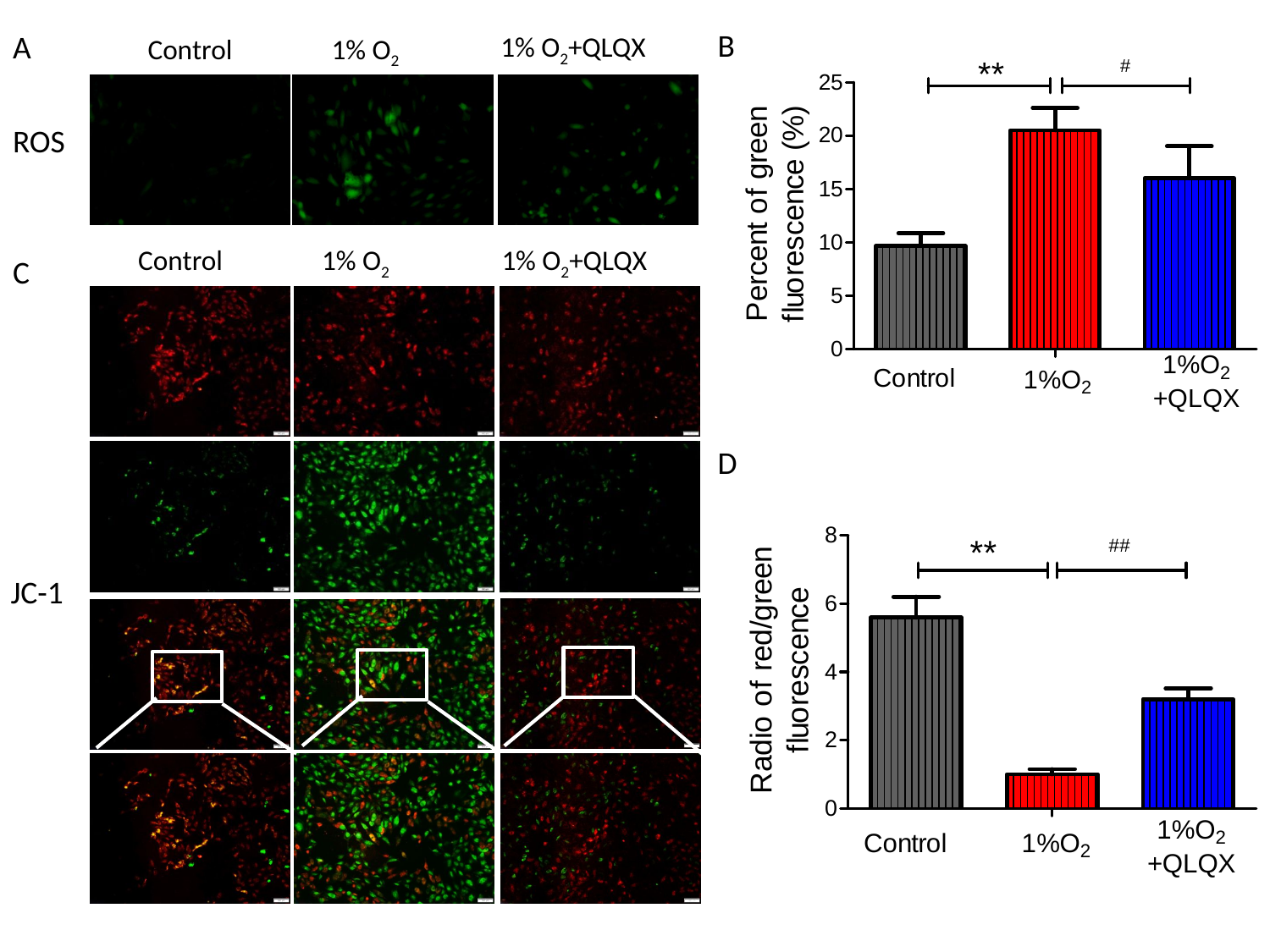

B
A
1% O2+QLQX
Control
1% O2
ROS
Control
1% O2
1% O2+QLQX
C
D
JC-1

## Slide 6
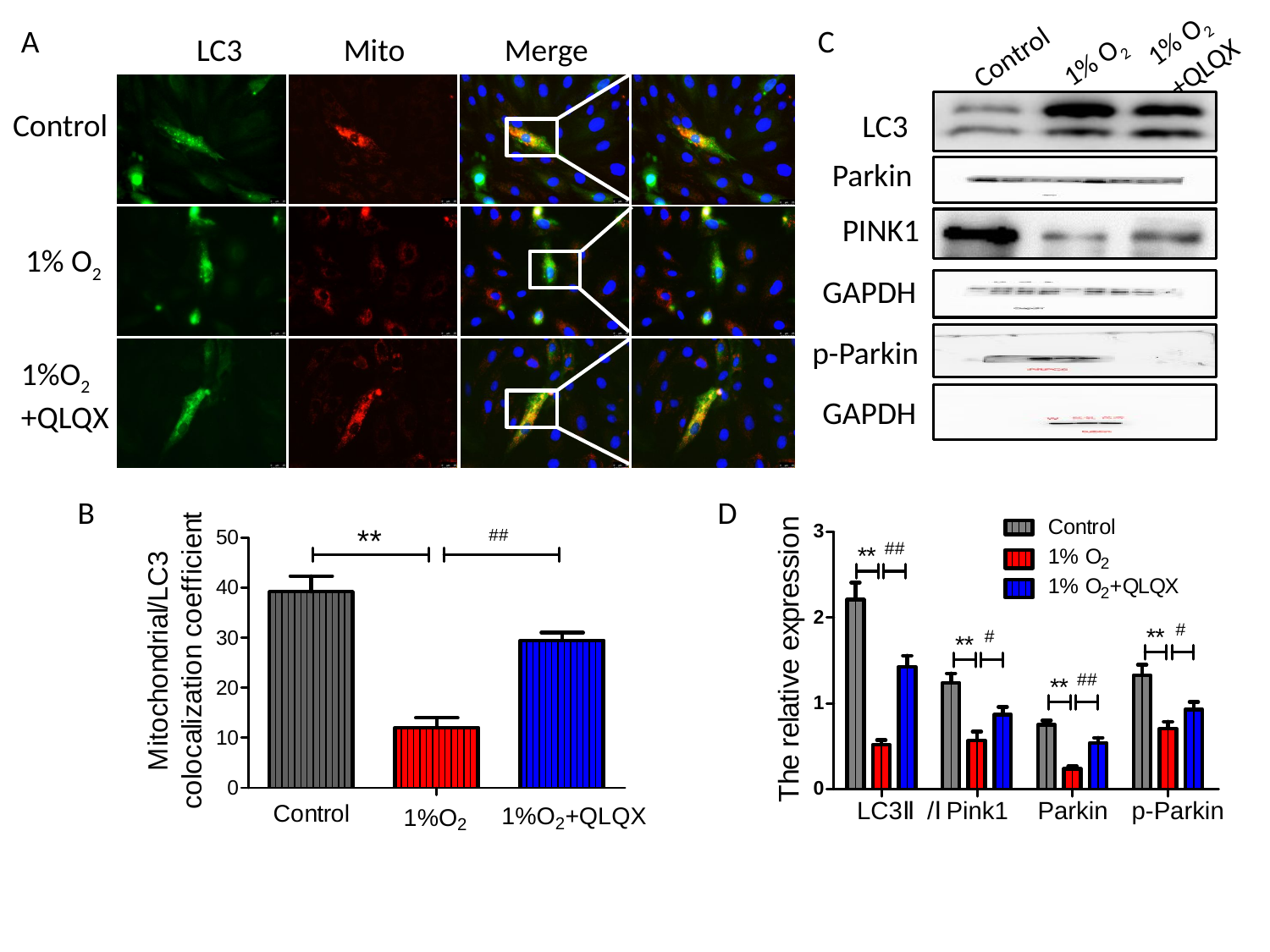

1% O2
+QLQX
1% O2
Control
LC3
Parkin
PINK1
GAPDH
p-Parkin
GAPDH
A
C
LC3
Mito
Merge
Control
1% O2
1%O2
+QLQX
B
D

## Slide 7
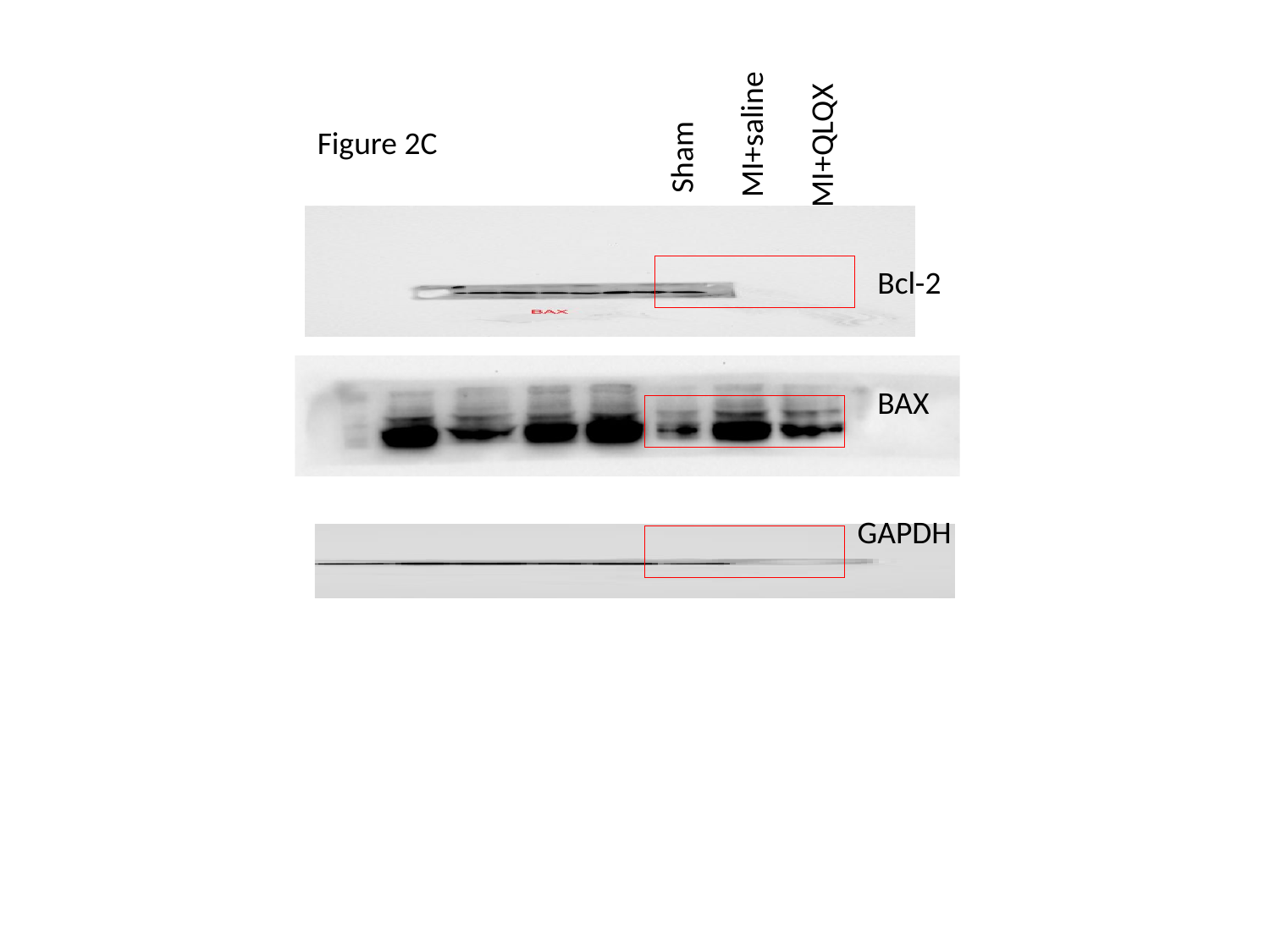

MI+saline
MI+QLQX
Figure 2C
Sham
Bcl-2
BAX
GAPDH

## Slide 8
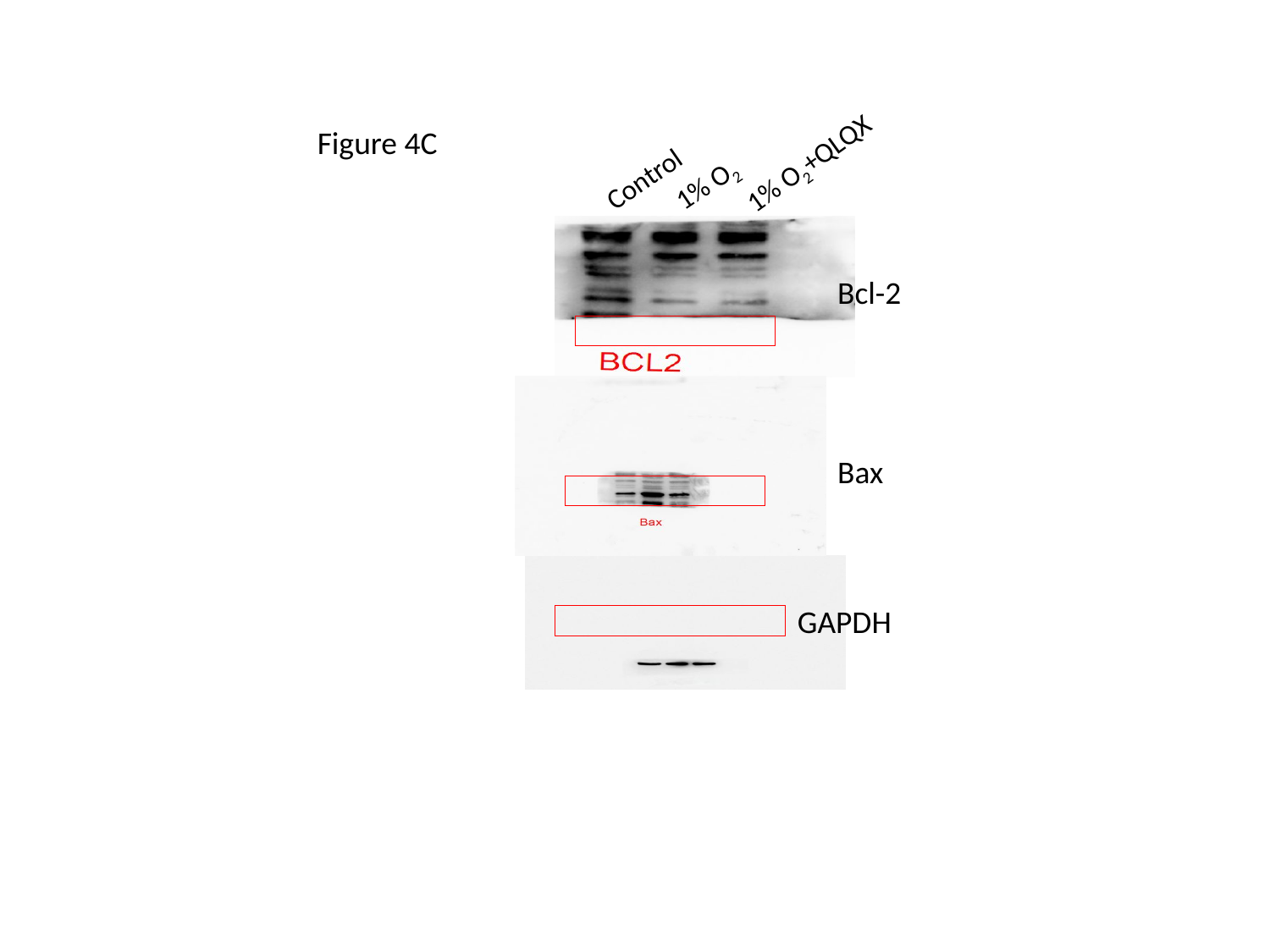

Figure 4C
1% O2+QLQX
1% O2
Control
Bcl-2
Bax
GAPDH

## Slide 9
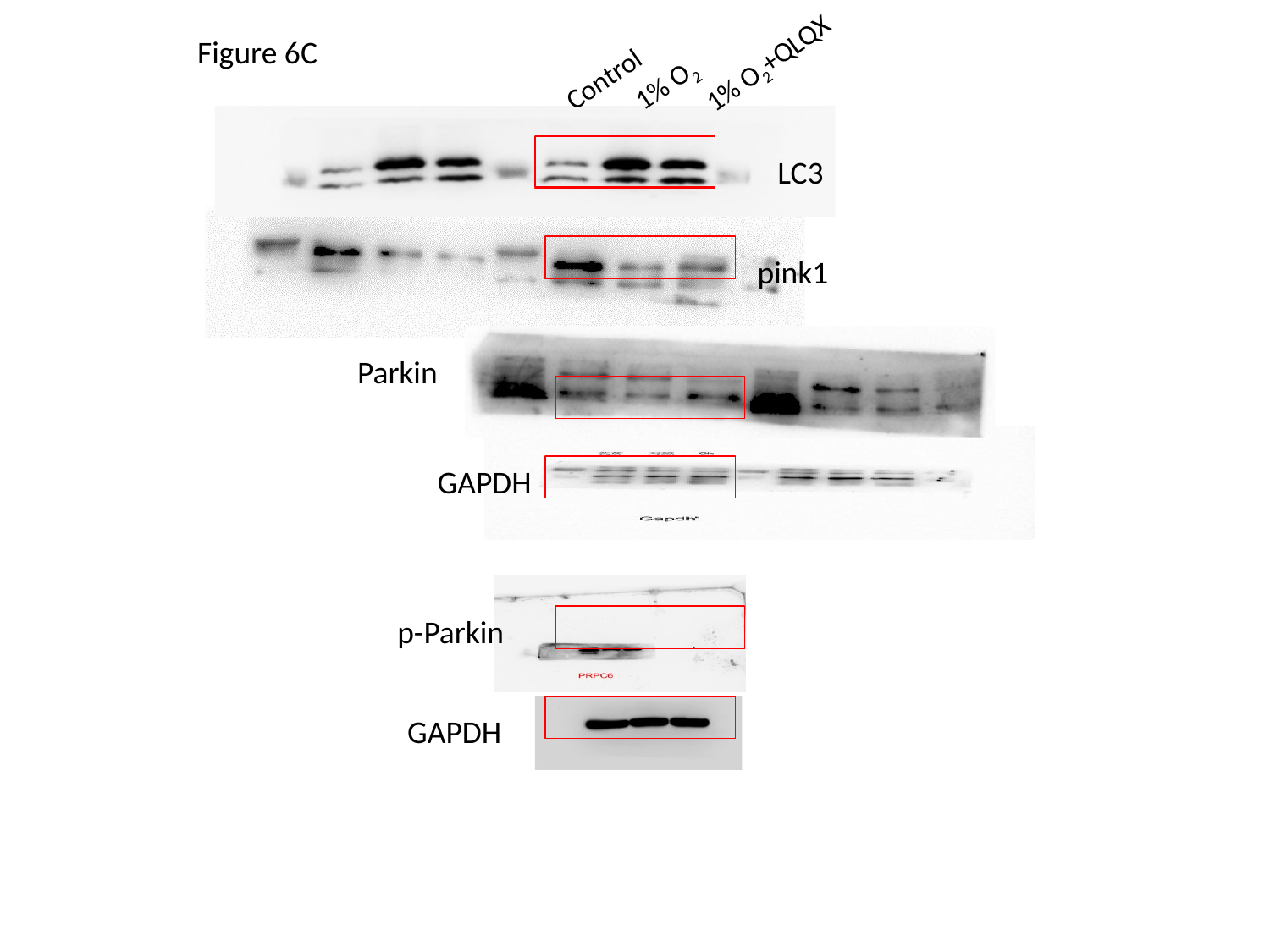

Figure 6C
1% O2+QLQX
1% O2
Control
LC3
pink1
Parkin
GAPDH
p-Parkin
GAPDH
