# Supplementary material for: Qiliqiangxin reduced cardiomyocytes apotosis and improved heart function in infarcted heart through Pink1/Parkin -mediated mitochondrial autophagy
Source: BMC Complement Med Ther. 2020 Jul 2;20:203. doi: 10.1186/s12906-020-02992-7 (PMC7330946; doi:10.1186/s12906-020-02992-7)
Supplement: Supplementary file 2 — Additional file 2. [file 12906_2020_2992_MOESM2_ESM.pdf]

江苏省实验动物质量合格证

No. 32002100001081

购买单位： 湖北医药学院附属太和医院　　　　动物实验单位： 湖北医药学院

动物品种	品系	等级	动物规格			数量
			体重	日龄	性别	
小鼠	FVB	SPF级	/	56	雌性	20
小鼠	FVB	SPF级	/	56	雄性	10
最近一次质量检测日期	2016年07月20日			质量检测单位	NBRI	
用途	科学研究			实验单位使用许可证编号	SYXK（鄂）2016-0031	
出售单位(盖章)	南京大学一南京生物医药研究院			许可证号	SCXK(苏)2015-0001	

质量负责人： 李灵恩　　　　经手人： 董俊　　　　日期： 2016年09月08日

开具使用证明
