# Supplementary material for: Qiliqiangxin reduced cardiomyocytes apotosis and improved heart function in infarcted heart through Pink1/Parkin -mediated mitochondrial autophagy
Source: BMC Complement Med Ther. 2020 Jul 2;20:203. doi: 10.1186/s12906-020-02992-7 (PMC7330946; doi:10.1186/s12906-020-02992-7)
Supplement: Supplementary file 3 — Additional file 3. [file 12906_2020_2992_MOESM3_ESM.docx]

Dear Editor,

I am very glad to receive comments from your and reviewers to our manuscript "Qiliqiangxin reduced cardiomyocytes apotosis and improved heart function in infarcted heart through Pink1/Parkin -mediated mitochondrial autophagy" (BCAM-D-19-01203R2) 。

We are thankful to the editor and the reviewers for pointing out some important modifications needed in the paper. We have thoughtfully taken into account these comments.

The new changes are marked in red, we appreciate for editors/reviewers’ warm work earnestly and hope that all these changes fulfill the requirements to make the manuscript acceptable for publication in BMC Complementary Medicine and Therapies.

Looking forward to hearing from you soon.

With best wishes,

Yours sincerely,

Junyang Zhou

Response to editor:

1. You can find the images of the original whole membranes, in the supplementary materials, and they are clearly labelled.

2. A point-by-point response letter was offered in the supplementary materials and reviewers can read it below. This letter provide a detailed response to each reviewer/editorial point raised.

3. We confirm Yan Ding as only one corresponding author in accordance with your requirements. We ensure that the corresponding author provided on the title page matches in the manuscript file and in the editorial manager system.
4. All changes to the manuscript are marked in red, you can find them easily.

Best wishes,

Junyang Zhou

Response to reviewers

**To reviewer 1**: No further response.

**To reviewer 2:**

1. In all legends to Figures authors must include the statistical methods used to compare the treatments, although the methods are described in methodology section.

**Response:** Thanks for your good suggestion. We have added statistical methods in all legends to Figures. Please see the revised manuscript.

3. References must be corrected in accordance to Journal style. As the references are written they do not follow the journal´s style:
Article within a journal

Smith JJ. The world of science. Am J Sci. 1999;36:234-5.
Article within a journal (no page numbers)
Rohrmann S, Overvad K, Bueno-de-Mesquita HB, Jakobsen MU, Egeberg R, Tjønneland A, et al. Meat consumption and mortality - results from the European Prospective Investigation into Cancer and Nutrition. BMC Medicine. 2013;11:63.

**Response:** References have corrected in accordance to Journal style. The citation was inserted by NoteExpress.

**To reviewer 3:** No further response.

**To reviewer 4:** No further response.

**To reviewer 5:** Overall the revised manuscript looks better. The introduction is however significantly lacking and need to be expanded with at least a paragraph that introduces the journal readers to the general health-promoting effect of herbal medicine. The following references could help in such a proposed paragraph: PMID: 26522014, PMID: 25584615, PMID: 27880817, PMID: 30134897, PMID: 30446676, PMID: 26260004, PMID: 31027364, DOI:10.1007/978-3-319-53880-8_3. After such revisions, I believe the manuscript would be ready for further processing and consideration.

**Response:** Thanks for your good suggestions. We have read all the recommended literature and put them in the background section (e.g. Background section, page 2, the third paragraph，line 1). In the background, we added the general health-promoting effect of Qiliqiangxin (e.g. Background section, page 2, the third paragraph, line 2 to 4).and its research progress in recent years. The specific components of QLQX are showed in Methods section (e.g. Methods section, page 3, the first paragraph, line 3 to 6). Please check it.

**Reviewer 6:** This is the first time that I evaluate this manuscript. Overall, this is a very interesting study to explore the role of QLQX in myocardial infarction with a focus on mitophagy mediated by Parkin. Although the authors have made three-rounds revision, some points should be addressed before publication:
1. In Fig.1, there is ~20% reduction in myocardial infraction which is evaluated through INF/AAR. Interestingly, the value of EF is improved about ~10 after treatment with QLQX. Therefore, the authors should carefully measure the "myocardial infarction area" because infarcted size correlates with cardiac function (EF%).

**Response:** Thank you very much for your good suggestion, we have remeasured the "myocardial infarction area", and updated relevant data. You can find it in Results section (e.g. Results section, page 6, the third paragraph, line 5 to 7).

2. In Fig.3, it is hard to observe the autophagosome by TEM. The author should use arrows to indicate where the autophagosome is.

**Response:** We have used red arrows to indicate location of the autophagosome, pleased see revised figure 3.
3. Although the upregulation of PINK and Parkin at protein levels may be a marker of mitophagy activation. However, several researches argue that phosphorylation parkin is required for mitophagy. Accordingly, the reviewer suggests the authors to add additional study to explore the alterations of phosphorylated Parkin in response to MI and QLQX.

**Response:** Thank you very much for your good suggestion, added the immunohistochemistry blot of p-parkin in Figure 3.The relevant data can be seen in Results section (e.g. Results section, page 7, the first paragraph, line 4 to 8).

4. In Fig.4, blots of Bax downregulation are inconsistent with the quantification analysis. The author should replace the representative picture.

**Response:** Thank you very much for your good suggestion, we have replace Bax with new picture in fig 4.

5. Similar concern to the alterations of PINK1 in Fig.5.

**Response:** Thank you very much for your good suggestion, we have updated the blot of PINK1 and added p-parkin blot in figure 6.

6. Discussion should include the following references: PMID: 30120595; PMID: 30310998; PMID: 27174950；PMID：29540794.

**Response:** We have downloaded these four articles and browsed them, these articles are very instructive, we have added them in the discussion (e.g. references, 29,26,32,33). We believe that these literatures will also have a good guiding effect on my future experiments.
